# *In vitro* Suppression of SARS-CoV-2 Infection by Existing Kampo Formulas and Crude Constituent Drugs Used for Treatment of Common Cold Respiratory Symptoms

**DOI:** 10.3389/fphar.2022.804103

**Published:** 2022-03-29

**Authors:** Masaki Kakimoto, Toshihito Nomura, Tanuza Nazmul, Hiroki Kitagawa, Keishi Kanno, Keiko Ogawa-Ochiai, Hiroki Ohge, Masanori Ito, Takemasa Sakaguchi

**Affiliations:** ^1^ Department of General Internal Medicine, Hiroshima University Hospital, Hiroshima, Japan; ^2^ Department of Infectious Disease, Hiroshima University Hospital, Hiroshima, Japan; ^3^ Department of Virology, Graduate School of Biomedical and Health Sciences, Hiroshima University, Hiroshima, Japan; ^4^ Kampo Clinical Center, Department of General Internal Medicine, Hiroshima University Hospital, Hiroshima, Japan

**Keywords:** anti-viral, COVID-19, SARS-CoV-2, herbal medicine, kampo medicine, crude drug

## Abstract

Several traditional Japanese Kampo formulas are known to have inhibitory effects on infections with viruses that cause respiratory symptoms. Although some herbs and their components have been reported to suppress SARS-CoV-2 replication *in vitro*, it is difficult to compare effective Kampo formulas because of the different methods used in studies. Thus, we carried out *in vitro* experiments on the suppression of SARS-CoV-2 infection by Kampo formulas and crude drugs used for the common cold to compare their suppressive effects on virus infection. After infecting VeroE6/TMPRSS2 cells with SARS-CoV-2, lysates of the Kampo formulas and crude drugs were added, and after 24 h, the infectious titer in the medium was measured by the TCID_50_ method. Maoto was the most effective among the Kampo formulas, and Ephedrae herba was the most effective among the constituent crude drugs. However, a comparison of the suppressive effects of Ephedrae herba and Kampo formulas containing Ephedrae herba showed that the suppressive effect on virus infection did not depend on the content of Ephedrae herba. Based on the results, we believe that the use of Maoto among Kampo formulas is suitable as a countermeasure against COVID-19.

## Introduction

In September 2021, the cumulative number of SARS-CoV-2 infection cases worldwide was more than 200 million and the number of deaths from SARS-CoV-2 infection was more than 4.5 million. There has been no apparent reduction in the number of new infections or deaths (World Health Organization, 2021). Although the efficacy of vaccines has been recognized and vaccination has been accelerated in many countries worldwide, there are still many unclear issues about vaccines as the efficacy of vaccines against emerging mutant strains (Liu et al., 2021). On the other hand, there are few drugs that can be expected to have antiviral effects and most of the drugs are expensive (Hsu, 2020). Therefore, there is an urgent need to find new anti-viral drugs or alternative treatments with drug repositioning.

Kampo medicine is a systematized medical system based on traditional East-Asian medicine and unique drugs. Kampo medicine originated in traditional Chinese medicine, which was introduced to Japan around the 5th century and was refined from the 17th century to the currently used Kampo medicine in Japan. Kampo formulas are mixtures of crude drugs, which are extracts of herbs, insects, minerals, fungi, and other substances (Tsumura and Co., 2016). All Kampo formulas were approved as drugs for humans by Japan’s Ministry of Health, Labour and Welfare in 1986 and are regulated by the National Institute of Health and Welfare (STORK, 2020). Among the medications certified by the Ministry of Health, Labour and Welfare, medical Kampo formulas are comparatively inexpensive, and many of them have versatility in use. On the other hand, herbal medicines themselves are also used as folk remedies not only in Japan but also in many other countries.

Influenza and respiratory syncytial viruses are RNA viruses and the primary symptoms of infections with these viruses are respiratory symptoms that are similar to the symptoms of SARS-CoV-2 infection. In Japan, two Kampo prescriptions, Maoto and Saikokeishito, have been approved for treatment of influenza infection. There have been *in vitro* studies showing suppression of infection with these viruses by Kampo formulas and crude drug extracts (Mantani et al., 1999; Nomura et al., 2019; Hou et al., 2020). There seems to be a relationship between the clinical effects of Kampo formulas and inhibition of influenza virus growth in cultured cells.

In this study, we examined the suppressive effects of Kampo formulas on SARS-CoV-2 infection using VeroE6/TMPRSS2 cells, which are highly susceptible to SARS-CoV-2 infection (Matsuyama et al., 2020). We selected eight Kampo formulas that have been shown to be effective against influenza virus infections and common cold symptoms and examined their inhibitory effects on SARS-CoV-2 infection. We also investigated the inhibitory effects of six crude constituent drugs constituting those Kampo formulas on SARS-CoV-2 infection.

## Materials and Methods

### Cells and Viruses

VeroE6/TMPRSS2 cells [African green monkey kidney-derived cells expressing human TMPRSS2, purchased from Japanese Collection of Research Bioresources (JCRB) Cell Bank, JCRB 1819] were propagated in Dulbecco’s modified Eagle’s minimum essential medium (DMEM, Invitrogen) supplemented with 10% fetal calf serum (FCS; Biosera, Kansas City, MO, United States), penicillin G (100 units/ml, Meiji Seika Pharma, Tokyo, Japan), and streptomycin (100 μg/ml, Meiji Seika Pharma). The cells were cultured at 37°C in 5% CO_2_. SARS-CoV-2/JP/Hiroshima-46059T/2020 (Yamamotoya et al., 2021; B1.1.1, GISAID accession ID: EPI_ISL_6289932, GenBank/DDBJ/EMBL accession number: MZ853926) was used as the test virus.

To prepare virus suspensions, VeroE6/TMPRSS2 cells were infected with the virus and incubated in DMEM. When cytopathic effects were fully developed, the culture supernatant was harvested and filtered through a 0.45-µm filter after low-speed centrifugation. The virus titer was determined by the standard 50% tissue culture infectious dose (TCID_50_) method. Briefly, a 10-fold serial dilution of the virus was inoculated into cells in a 96-well plate in tetraplicate or octuplicate and incubated for 7 days to check for CPE. Based on this result, infectivity was calculated and expressed as TCID_50_/ml, as described previously (Nomura et al., 2021).

### Reagents

Extract powders of Kampo formulas including Maoto, Saikokeishito, Shomakakkonto, Kakkonto, Shoseiryuto, Senkyuchachosan, Bakumondoto, and Hochuekkito were kindly provided by Tsumura & Co. (Tokyo, Japan) (Table 1). Crude drugs including *Glycyrrhiza uralensis* Fisch. ex DC [Fabaceae] (Glycyrrhizae radix), *Ephedra sinica* Stapf [Ephedraceae] (Ephedrae herba), *Paeonia lactiflora* Pall [Paeoniaceae] (Paeoniae radix), *Ligusticum officinale* (Makino) Kitag [Apiaceae] (Cnidii rhizoma), *Scutellaria baicalensis* Georgi [Lamiaceae] (Scutellariae radix), and *Bupleurum falcatum* L [Apiaceae] (Bupleuri radix) were purchased from Tsumura & Co. The Kampo formulas used in this study and their constituent crude drugs (generic name, scientific name, and percentage included) are shown in detail in Table 1.

**TABLE 1:** The Kampo medicines used in this study and their constituent crude drugs.

**Maoto**																						
Indications	Common cold, Influenza (Acute), Rheumatoid arthritis																					
Crude drugs (Latin name)	Armeniacae semen							Ephedrae herba^a^								Cinnamomi cortex					Glycyrrhizae radix*	
Scientific name	*Prunus armeniaca var. armeniaca [Rosaceae] Prunus armeniaca* L*. [Rosaceae]*							*Ephedra sinica* Stapf *[Ephedraceae]*								*Neolitsea cassia (*L.) *Kosterm. [Lauraceae]*					*Glycyrrhiza uralensis* Fisch. ex DC*. [Fabaceae]*	
Rate of crude drugs configuring Kampo extracts (excluding additives)	32.3%							32.3%								25.8%					9.7%	
**Saikokeishito**																						
Indications	Common cold, Influenza, Feverish diseases such as pneumonia and pulmonary tuberculosis																					
	Gastric ulcer, Duodenal ulcer, Cholecystitis, Gallstone, Pain in liver dysfunction and pancreatitis																					
Crude drugs (Latin name)	Bupleuri radix^a^	Pinelliae Tuber			Scutellariae radix^a^			Glycyrrhizae radix^a^		Cinnamomi cortex		Paeoniae radix^a^				Ziziphi fructus	Ginseng radix				Zingiberis rhizoma —	
Scientific name	*Bupleurum falcatum* L*. [Apiaceae]*	*Pinellia ternata (*Thunb.) Makino *[Araceae]*			*Scutellaria baicalensis* Georgi *[Lamiaceae]*			*Glycyrrhiza uralensis* Fisch. ex DC*. [Fabaceae]*		*Neolitsea cassia* (L.) Kosterm. *[Lauraceae]*		*Paeonia lactiflora* Pall. *[Paeoniaceae]*				*Ziziphus jujuba* Mill. *[Rhamnaceae]*	*Panax ginseng* C.A.Mey. *[Araliaceae]*				*Zingiber officinale* Roscoe *[Zingiberaceae]* *—*	
Rate of crude drugs configuring Kampo extracts (excluding additives)	22.7%	18.2%			9.1%			9.1%		9.1%		9.1%				9.1%	9.1%				4.6% —	
**Kakkonto**																						
Indications	Common cold, Early stage of febrile disease, Inflammatory diseases (conjunctivitis, keratitis, otitis media, tonsillitis, mastitis, lymphadenitis)																					
	Stiff shoulders, Neuralgia in the upper body, Urticaria																					
Crude drugs (Latin name)	Puerariae Radix	Ziziphi fructus			Ephedrae herba^a^			Glycyrrhizae radix^a^			Cinnamomi cortex				Paeoniae radix^a^				Zingiberis rhizoma			
Scientific name	*Pueraria montana var. lobata* (Willd.) Maesen & S.M.Almeida ex Sanjappa & Predeep *[Fabaceae]*	*Ziziphus jujuba* Mill. *[Rhamnaceae]*			*Ephedra sinica i[Ephedraceae]*			*Glycyrrhiza uralensis* Fisch. ex DC. *[Fabaceae]*			*Neolitsea cassia* (L.) Kosterm. *[Lauraceae]*				*Paeonia lactiflora* Pall. *[Paeoniaceae]*				*Zingiber officinale* Roscoe *[Zingiberaceae]*			
Rate of crude drugs configuring Kampo extracts (excluding additives)	22.2%	16.7%			16.7%			11.1%			11.1%				11.1%				11.1%			
**Shomakakkonto**																						
Indications	Common cold (early stage), Dermatitis																					
Crude drugs (Latin name)	Puerariae Radix			Paeoniae radix^a^			Cimicifugae Rhizoma							Glycyrrhizae radix^a^						Zingiberis rhizoma		
Scientific name	*Pueraria montana var. lobata* (Willd.) Maesen & S.M.Almeida ex Sanjappa & Predeep *[Fabaceae]*			*Paeonia* lactiflora Pall. [Paeoniaceae]			*Actaea dahurica (*Turcz. ex Fisch. & C.A.Mey.*) Franch. [Ranunculaceae]・ Actaea heracleifolia* (Kom.) *J.Compton [Ranunculaceae]・ Actaea cimicifuga* L. *[Ranunculaceae]*							*Glycyrrhiza uralensis* Fisch. ex DC. *[Fabaceae*]						*Zingiber officinale* Roscoe *[Zingiberaceae]*		
Rate of crude drugs configuring Kampo extracts (excluding additives)	41.7%			25.0%			16.7%							12.5%						4.2%		
**Shoseiryuto**																						
Indications	Common cold, Bronchitis, Bronchial asthma, Rhinitis, Allergic Rhinitis, Allergic conjunctivitis																					
Crude drugs (Latin name)	Pinelliae tuber	Paeoniae radix^a^			Zingiberid rhizoma processum			Glycyrrhizae radix^a^		Cinnamomi cortex		Asiasari radix				Schisandrae fructus				Ephedrae herba^a^		
Scientific name	*Pinellia ternata* (Thunb.) Makino *[Araceae]*	*Paeonia* lactiflora Pall. [Paeoniaceae]			*Zingiber officinale* Roscoe *[Zingiberaceae]*			Glycyrrhiza uralensis Fisch. ex DC. [Fabaceae]		*Neolitsea cassia* (L.) Kosterm. [Lauraceae]		*Asarum heterotropoides* F.Schmidt [Aristolochiaceae]				*Schisandra chinensis* (Turcz.) Baill. [Schisandraceae]				*Ephedra sinica* Stapf [Ephedraceae]		
Rate of crude drugs configuring Kampo extracts (excluding additives)	22.2%	11.1%			11.1%			11.1%		11.1%		11.1%				11.1%				11.1%		
**Senkyuchachosan**																						
Indications	Common cold, automatic imbalance syndrome peculiar to women resembling climacteric disturbance, and headache																					
Crude drugs (Latin name)	Cyperi rhizoma		Cnidii rhizoma^a^		Notopterygii rhizoma			Schizonepetae spica		Menthae herba		Angelicae dahuricae radix				Saposhnikoviae radix	Glycyrrhizae radix^a^				Camelliae sinensis folium —	
Scientific name	*Cyperus rotundus* L*. [Cyperaceae]*		*Ligusticum officinale (*Makino) Kitag*. [Apiaceae]*		*Hansenia weberbaueriana (*Fedde ex H.Wolff*)* Pimenov & Kljuykov *[Apiaceae]*			*Nepeta tenuifolia* Benth. *[Lamiaceae]*		*Mentha canadensis* L*. [Lamiaceae]*		*Angelica dahurica* (Hoffm.) Benth. & Hook*.f. ex* Franch*. & Sav. [Apiaceae]*				*Saposhnikovia divaricata (*Turcz. ex Ledeb.) Schischk. *[Apiaceae]*	*Glycyrrhiza uralensis* Fisch. ex DC. *[Fabaceae]*				*Camellia sinensis (*L.*) Kuntze [Theaceae]*	
Rate of crude drugs configuring Kampo extracts (excluding additives)	20.0%		15.0%		10.0%			10.0%		10.0%		10.0%				10.0%	7.5%				7.5%	
**Bakumondoto**																						
Indications	Coughing with a hard, Obstructive sputum, Bronchitis, and bronchial asthma																					
Crude drugs (Latin name)	Ophiopogonis radix		Oryzae fructus			Pinelliae tuber			Ziziphi fructus				Glycyrrhizae radix^a^					Ginseng radix				
Scientific name	Ophiopogon japonicus (Thunb.) Ker Gawl. [Asparagaceae]		Oryza sativa L. [Poaceae]			*Pinellia ternata (Thunb.) Makino [Araceae]*			Ziziphus jujuba Mill. [Rhamnaceae]				Glycyrrhiza uralensis Fisch. ex DC. [Fabaceae]					*Panax ginseng C.A.Mey. [Araliaceae]*				
Rate of crude drugs configuring Kampo extracts (excluding additives)	37.0%		18.5%			18.5%			11.1%				7.4%					7.4%				
**Hochuekkito**																						
Indications	Common cold, Summer thinness, Loss of appetite, Gastroptosis, Hemorrhoids, Anal prolapse, Drooping uterus, Pubic atrophy, Hyperhidrosis																					
Crude drugs (Latin name)	Astragali radix	Atractylodis Lanceae Rhizoma			Ginseng radix			Angelicae acutilobae radix		Bupleuri radix^a^		Ziziphi fructus				Aurantii nobilis pericarpium	Glycyrrhizae radix^a^				Cimicifugae Rhizoma	Zingiberis rhizoma
Scientific name	*Astragalus mongholicus* Bunge [Fabaceae]	*Atractylodes lancea* (Thunb.) DC. [*Asteraceae*]			*Panax ginseng* C.A.Mey. *[Araliaceae]*			*Angelica acutiloba (Siebold & Zucc.)* Kitag. *[Apiaceae]*		*Bupleurum falcatum* L. *[Apiaceae]*		*Ziziphus jujuba* Mill. *[Rhamnaceae]*				*Citrus deliciosa* Ten*. [Rutaceae]*	*Glycyrrhiza uralensis Fisch.* ex DC. *[Fabaceae]*				*Actaea dahurica (*Turcz. ex Fisch. & C.A.Mey.*) Franch. [Ranunculaceae]・ Actaea heracleifolia (*Kom*.) J.Compton [Ranunculaceae]・ Actaea cimicifuga* L*. [Ranunculaceae]*	*Zingiber officinale Roscoe [Zingiberaceae]*
Rate of crude drugs configuring Kampo extracts (excluding additives)	16.7%	16.7%			16.7%			12.5%		8.3%		8.3%				8.3%	6.3%				4.2%	2.1%

Kampo medicines used in this study and their constituent crude drugs are shown with indications of the Kampo medicines. The ratio of constituents included in Kampo drugs of extract powder and crude drugs referred to the website of Tsumura & Co.

a The crude drugs investigated are marked.

Solutions of the Kampo formulas for testing were prepared as described previously (Nomura et al., 2019). The powder of each Kampo formula was mixed with DMEM to a concentration of 20 mg/ml. The powder was dissolved at 50°C for 1 h and the mixture was centrifuged at a low speed and then the supernatant was filter-sterilized through a 0.22-μm filter. The crude drugs were prepared in a similar way. For both the Kampo formulas and the crude drugs, little insoluble material was found after low-speed centrifugation, and the weight of the initial powder was therefore used as the weight of the solute.

### Cytotoxicity Assay

VeroE6/TMPRSS2 cells were cultured in DMEM with the specified concentrations of reagents for 24 h, and lactate dehydrogenase (LDH), which was released from the cells into the medium, was assayed with a colorimetric method using the Cytotoxicity LDH Assay Kit-WST (Dojindo Laboratories, Kumamoto, Japan) by measuring absorbance at 490 nm in the TriStar LB 941 plate reader (Bertohold Technologies, Wildbad, Germany). The cytotoxicity of the reagents was calculated from the absorbance measurements as 100% for the high control (cell lysis by surfactant) and 0% for the low control (culture medium only).

### Replication of SARS-CoV-2 *in vitro*

Confluent monolayers of VeroE6/TMPRSS2 cells in a 96-well plate were infected with 50 µl/well of the virus at an input multiplicity of infection (m.o.i.) of 0.05 or 10. After adsorption for 2 h, the inoculated viruses were removed, and the cells were further cultured in 100 µl/well of DMEM containing different concentrations of Kampo formulas or crude drugs. The conditions of m.o.i. and virus adsorption time were based on the conditions used in a previous study for effective infection of cells (Wang et al., 2020). The medium was harvested after 24 h, and viral infectivity was assayed by the TCID_50_ method. The logarithm of infectivity titer and reagent concentration in the medium were plotted and an approximation straight-line was drawn to calculate the 50% inhibitory concentration (IC_50_), as described previously (Nomura et al., 2021).

We conducted infection experiments under two conditions: m o.i. of 0.05 and m.o.i. of 10. At m.o.i. of 10, all cells are infected at once, allowing us to observe the process of virus entry and replication (one-step replication). When m.o.i. of 0.05, on the other hand, 20 cells are infected with a single virus. In addition to the entry and replication of the virus into the cell, the progeny virus is released from the cell and further infects the surrounding cells (multi-step replication).

### Assay for Inactivation of Viral Particles

For the Kampo formulas, the solution was mixed with 90 µl of the drug at a concentration of 20 mg/ml and 10 µl of the virus solution at 2.0 × 10^9^ TCID_50_/ml and incubated for 3 min at room temperature. The mixture was then serially diluted 10-fold in DMEM, and adsorbed on VeroE6/TMPRSS cells for 1 h. The inoculum was removed, and cell culture medium was added and incubated for 7 days. The infectivity of the solution was determined by the TCID_50_ method. For crude drugs, reagents at 1.25–10 mg/ml concentrations were used considering their cytotoxicity. Phosphate-buffered saline (PBS) was used as an untreated control, and 70% (w/w) ethanol was used as an inactivation control as described previously (Nomura et al., 2021).

## Results

### Suppressive Effects of Kampo Formulas on SARS-CoV-2 Infection

Eight Kampo formulas used to treat respiratory symptoms, including respiratory symptoms of influenza and common cold infections, were investigated for their inhibitory effects on SARS-CoV-2 infection *in vitro*. Table 1 shows the clinical indications of each Kampo formula, the crude drugs included, and the proportions of the crude drugs.

**FIGURE 1:**
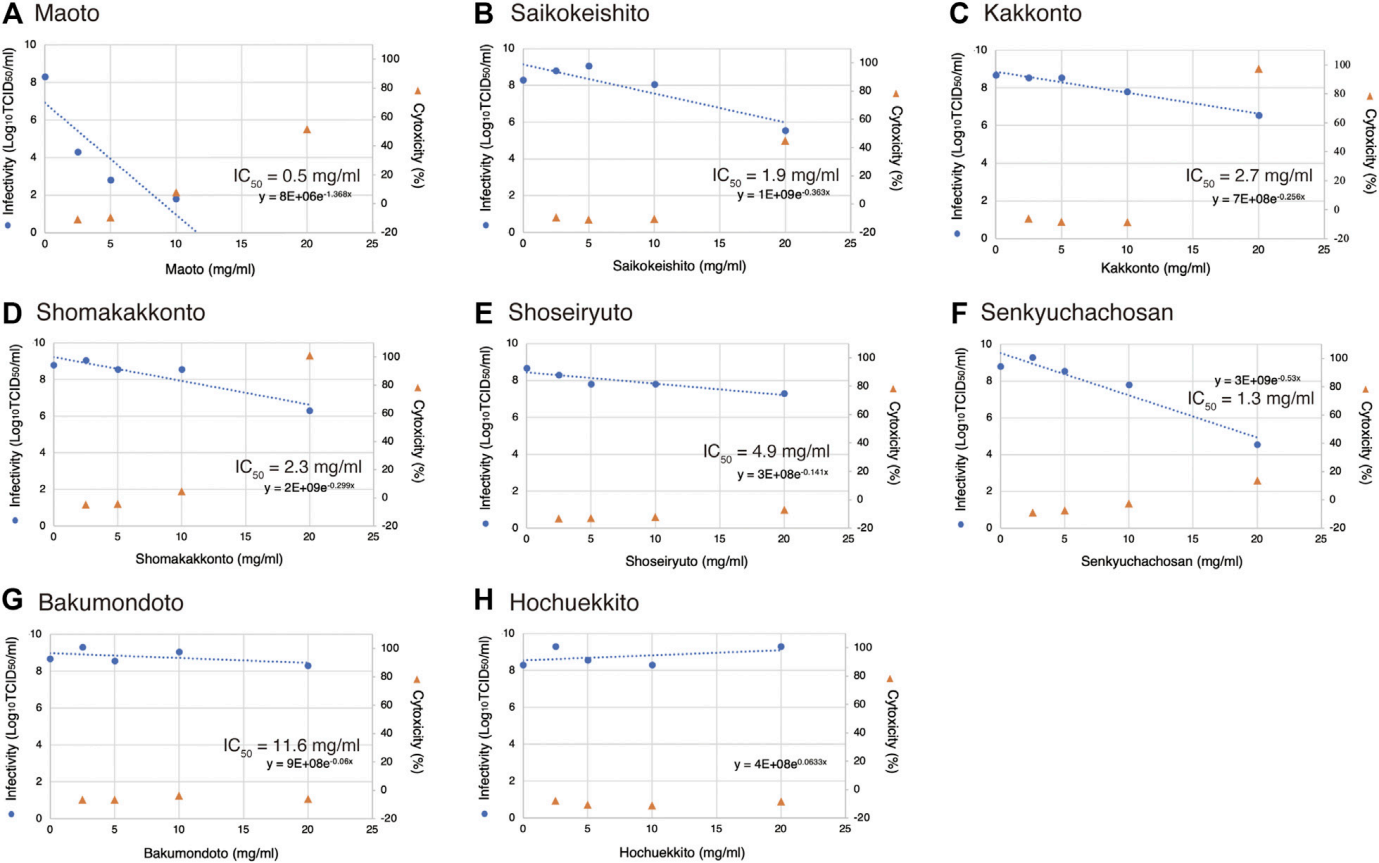
Inhibition of SARS-CoV-2 replication by Kampo formulas. **(A-H)** The Kampo formula used is noted at the top of each panel. VeroE6/TMPRSS2 cells were infected with SARS-CoV-2 at an m.o.i. of 0.05. After 2-h adsorption, the inoculum was removed and the cells were cultured in DMEM containing different concentrations of Kampo formulas for 24 h. The viral infectivity in the medium was assayed by the TCID_50_ method. The left y-axis of the graph is the infectivity for each concentration. An exponential approximation was made by Excel to calculate the drug concentration that reduces the infectivity in the absence of a drug to 50% (IC_50_). The IC_50_ values and approximation equations are shown in the graph. If there is no decrease, the IC_50_ is not shown (Panel 1). VeroE6/TMPRSS2 cells were incubated in DMEM supplemented with the designated concentrations of a Kampo formula for 24 h, and LDH values in the media were then measured to evaluate cytotoxicity. The LDH value from detergent-treated cells was set at 100%, and the right y-axis is the percent inhibition of cytotoxicity for each concentration.

Initially, the cytotoxicity of each Kampo formula was examined by an LDH assay, and the values were plotted on a graph (Figure 1, △ marker, right Y-axis). Twenty mg/ml of each Kampo formula of Maoto (Figure 1A), Saikokeishito (Figure 1B), Kakkonto (Figure 1C), and Shomakakkonto (Figure 1D) showed more than 30% cytotoxicity. However, at concentrations below 10 mg/ml, none of the Kampo formulas showed apparent cytotoxicity. The results at concentrations below 10 mg/ml could be interpreted without considering the effect of cytotoxicity, while at 20 mg/ml, the results should be interpreted with caution.

VeroE6/TMPRSS2 cells were infected with SARS-CoV-2 at an m.o.i. of 0.05, and each Kampo formula was added to the medium. After 24 h, the infection titer was measured by the TCID_50_ method and plotted on a graph against the concentration of the Kampo formula (Figure 1, ○ marker, left Y-axis). In the case of Maoto, viral replication was inhibited as the concentration was increased and was almost completely inhibited at a concentration of 10 mg/ml (Figure 1A). An approximate line of these points was drawn, and the equation is shown in the graph. The 50% inhibitory concentration (IC_50_) that was calculated from the equation is also shown in the graph (Figure 1A). In the case of Hochuekkito, there was little change even when the drug concentration was increased (Figure 1H). According to the IC_50_ data shown in Figure 1, both Maoto and Senkyuchachosan had strong suppressive effects on SARS-CoV-2 infection. On the other hand, no suppressive effects of Bakumondo and Hochuekkito on virus infection were observed.

The cytotoxicity of Saikokeishito at 20 mg/ml was 44.7%, and we cannot deny the possibility that its suppressive effect on virus infection is due to its cytotoxicity. Since Kakkonto and Maoto showed even higher cytotoxicity at a concentration of 20 mg/ml, it was difficult to clearly determine their suppressive effects on virus infection at that concentration. Still, it can be considered that they have clear suppressive effects on virus infection at concentrations below 10 mg/ml.

**FIGURE 2:**
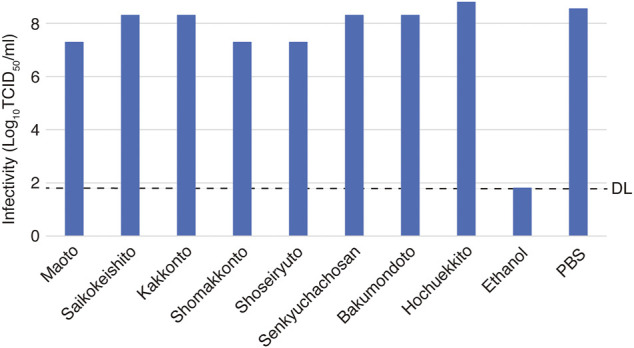
Effects of Kampo formulas on virus particle inactivation. A solution of the Kampo formula (20 mg/ml, 90 µl) and 10 µl of the virus solution at 2.0 × 10^9^ TCID_50_/ml was incubated for 3 min at room temperature. The mixture was then serially diluted 10-fold in DMEM, and the infectivity was determined by the TCID_50_ method. Phosphate-buffered saline (PBS) was used as an untreated control, and ethanol [70% (w/w)] was used as an inactivation control. The dotted line indicates the detection limit (DL) of the infectivity assay.

When the highest concentration (20 mg/ml) of each Kampo formula was mixed with the virus and the infectious titer was measured, none of the Kampo formulas decreased the infectious titer. This suggests that there was no direct inactivating effect of each Kampo formula on the virus particles (Figure 2).

In this experiment, the virus was inoculated into cells at an m.o.i. of 0.05. If the virus is inhibited by the Kampo formula, then the virus may be suppressed at one of the following stages: intracellular multiplication, release from the cell, or reinfection of neighboring cells. From the results of the experiment described above (Figure 2), it is unlikely that the viral particles are directly inactivated, and intracellular proliferation or release from the cell may therefore be impaired.

### Suppressive Effects of Crude Drugs on SARS-CoV-2 Infection

**FIGURE 3:**
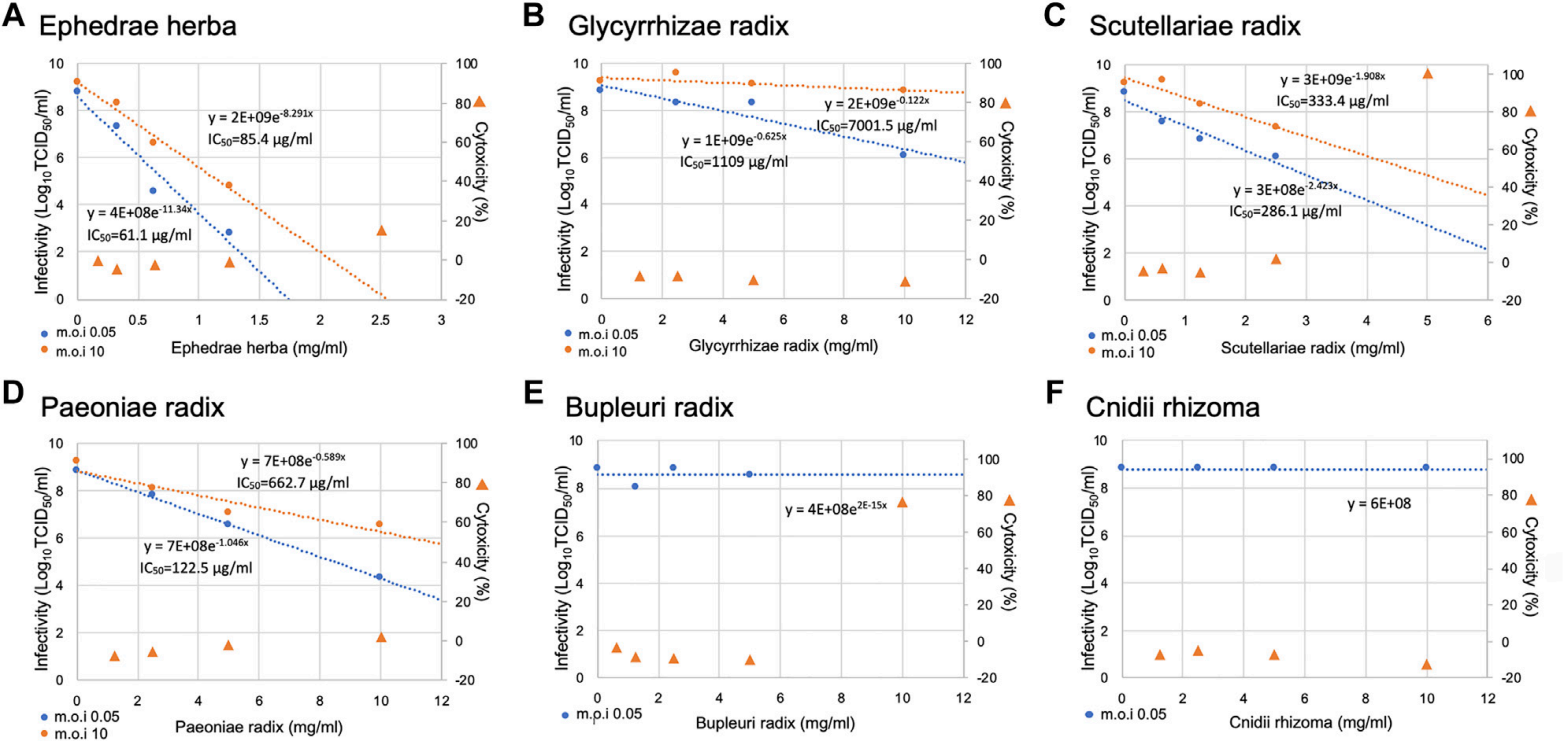
Inhibition of SARS-CoV-2 replication by crude drugs. **(A-F)** The crude drug used is noted at the top of each panel. VeroE6/TMPRSS2 cells were infected with SARS-CoV-2 at an m.o.i. of 0.05 or 10. After 2-h adsorption, the inoculum was removed and the cells were cultured in DMEM containing different concentrations of crude drugs for 24 h. The viral infectivity in the medium was assayed by the TCID_50_ method. The left y-axis of the graph is the infectivity for each concentration. An exponential or linear approximation was made by Excel to calculate the drug concentration that reduces the infectivity in the absence of a drug to 50% (IC_50_). The IC_50_ values and approximation equations are shown in the graph. If there is little or no decrease, the IC_50_ is not shown **(E,F)**. VeroE6/TMPRSS2 cells were incubated in DMEM supplemented with the designated concentrations of a Kampo fomula for 24 h, and LDH values in the media were then measured to evaluate cytotoxicity. The LDH value from detergent-treated cells was set at 100%, and the right y-axis is the percent inhibition of cytotoxicity for each concentration.

We investigated the suppressive effects on virus infection of the available crude drugs with high percentages of composition among the crude drugs constituting Maoto, Senkyuchachosan, and Saikokeishito, which had strong inhibitory effects on SARS-CoV-2 infection. The crude drugs tested were Ephedrae herba, Glycyrrhizae radix, Scutellariae radix, Paeoniae radix, Bupleuri radix, and Cnidii rhizoma (Figure 3). Ephedrae herba showed cytotoxicity at concentration of 2.5 mg/ml and above, Bupleuri radix showed cytotoxicity at concentrations of 10 mg/ml and above, and Scutellariae radix showed cytotoxicity at concentrations of 5 mg/ml and above. Therefore, the effects of these crude drugs at lower concentrations were evaluated.

The cells were infected with the virus at an m.o.i. of 0.05, and the infectious titer was measured by the TCID_50_ method (Figure 3). Ephedrae herba showed a strong inhibitory effect on virus infection, while Paeoniae radix, Scutellariae radix, and Glycyrrhizae radix showed weaker inhibitory effects (Figure 3).

To investigate the mechanisms by which the crude drugs suppress virus infection, we conducted infection experiments using crude drugs (Ephedrae herba, Scutellariae radix, Paeoniae radix, Glycyrrhizae radix) at an m.o.i. of 10 so that all of the cells would be infected (Figure 3). Ephedrae herba showed a strong suppressive effect on virus infection even at an m.o.i. of 10, and Paeoniae radix and Scutellariae radix also showed suppressive effects (Figure 3). These results suggest that Ephedrae herba, Paeoniae radix, and Scutellariae radix act on virus-infected cells to inhibit viral replication in the cells. On the other hand, Glycyrrhizae radix showed little inhibitory effect in the infection experiment with an m.o.i. of 10 (Figure 3), suggesting that the antiviral effect of Glycyrrhizae radix is due to its inhibition of spread of the virus to neighboring uninfected cells.

**FIGURE 4:**
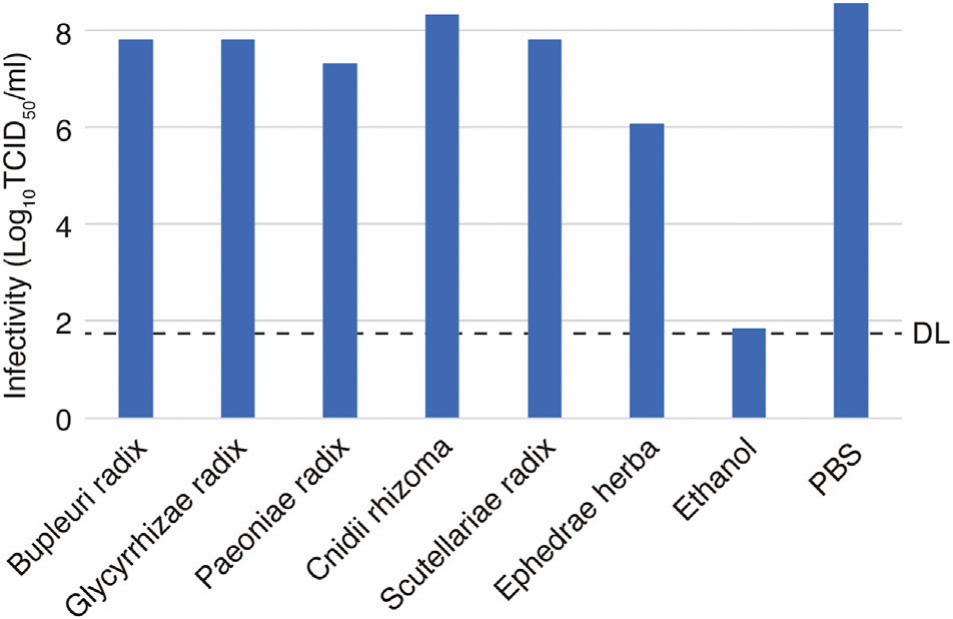
Effects of crude drugs on virus particle inactivation. A solution of a crude drug (90 µl) and 10 µl of the virus solution at 2.0 × 10^9^ TCID_50_/ml was incubated for 3 min at room temperature. Reagents at 1.25–10 mg/ml concentrations were used considering their cytotoxicity. The mixture was then serially diluted 10-fold in DMEM, and the infectivity was determined by the TCID_50_ method. Phosphate-buffered saline (PBS) was used as an untreated control, and ethanol [70% (w/w)] was used as an inactivation control. The dotted line indicates the detection limit (DL) of the infectivity assay.

The inactivating effect of each of the crude drugs on virus particles was investigated by mixing the crude drug at the highest concentration used in the virus infection experiment and measuring the infectivity titer (Figure 4). Ephedrae herba decreased the infections titer by 2.5 Log_10_ (TCID_50_/ml) compared to the control, suggesting that Ephedrae herba may act directly on the virus particles to inactivate them. The other crude drugs did not reduce the infectious titer, indicating that they did not inactivate the virus particles (Figure 4).

### Contribution of Epherae Herba in Kampo Formulas

In this study, we found that Ephedrae herba has a strong inhibitory effect on SARS-CoV-2 infection. Ephedrae herba is found in several Kampo formulas, and to verify the role of Ephedrae herba in inactivation of SARS-CoV-2 by the herbal medicines, we recalculated the effect of Ephedrae herba on SARS-CoV-2 based on the amount of Ephedrae herba in each Kampo formula.

**FIGURE 5:**
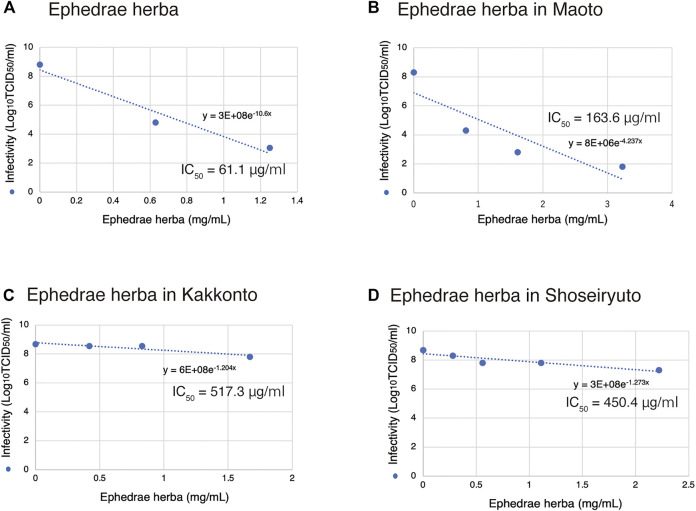
Inhibition of SARS-CoV-2 replication based on the amount of Ephedrae herba contained. **(A-D)** The origin of Ephedra herba is noted at the top of each panel. The results for growth inhibition of SARS-CoV-2 by Ephedrae herba and Kampo formulas containing Ephedrae herba (Figure 1) are re-plotted in a graph based on the amount of Ephedrae herba contained. An exponential approximation was made by Excel to calculate the drug concentration that reduces the infectivity in the absence of a drug to 50% (IC_50_). The IC_50_ values and approximation equations are shown in the graph.

The IC_50_ value of Ephedrae herba alone was 61.1 μg/ml (Figures 3A, 5A). Maoto contains 32.3% of Ephedrae herba (Table 1), and the IC_50_ value was calculated by plotting the amount of Ephedrae herba in Maoto on the horizontal axis of the graph (Figure 5B) to be 163.6 μg/ml (Figure 5B). Thus, although it should be the same amount of Ephedrae herba, Ephedrae herba in the form of Maoto was weakened, suggesting that other components of Maoto may be inhibiting the effect Ephedrae herba. The IC_50_ value of Maoto itself was calculated to be 0.5 mg/ml (Figure 1A), being consistent with this hypothesis.

Similarly, in the case of Kakkonto containing 16.7% of Ephedrae herba (Table 1), the IC_50_ value was calculated to be as high as 517.3 μg/ml based on the amount of Ephedrae herba (Figure 5C). The IC_50_ value of Kakkonto itself was 2.7 mg/ml (Figure 1C). Shoseiryuto contained 11.1% of Ephedrae herba (Table 1), and the IC_50_ value of Ephedrae herba was as high as 450.4 μg/ml based on Ephedrae herba (Figure 5D). The IC_50_ value of Shoseiryuto itself was 4.9 mg/ml (Figure 1E). These results suggest that the inhibitory effect of Ephedrae herba in the Kampo formulas on SARS-CoV-2 infection is weakened by other components.

## Discussion

Kampo formulas consist of various combinations of crude drugs, traditionally regarded as units, and are often used on the base of ancient experience (Tanaka et al., 1995; Odaguchi et al., 2019). In Japan, Kampo formulas are covered by the national health insurance system and are prescribed by doctors under the condition of government subsidies. Some Kampo formulas are also available as over-the-counter drugs that can be purchased at pharmacies without a doctor’s prescription. The extensive experience of using Kampo formulas in Japan has led to their safe and easy use in medical practice. In recent years, scientific analysis of the effects of Kampo formulas has provided evidence of their clinical benefits, and their clinical usefulness for the treatment of COVID-19 has also been suggested (Motoo et al., 2014; Takayama et al., 2021).

Some advantages of Kampo formulas are that they can be taken orally, are relatively inexpensive, and can be taken at an early stage. For example, Maoto, one of the Kampo formulas examined in this study, can be used to treat common colds in infants even before the onset of cold symptoms such as fever and nasal discharge (Kubo and Nishimura, 2007; Nagai et al., 2014). On the other hand, antivirals, as shown in clinical trials for influenza (Muthuri et al., 2014; Dobson et al., 2015), need to be taken after the onset of illness and before the peak of viral replication. The clinical course of COVID-19 is longer than that of influenza, and viral replication in patients with COVID-19 likely to continue for a long time. Although existing antivirals such as remdesivir may be effective if treatment with the antivirals started after the diagnosis of COVID-19, consideration should be given to the use of Kampo formulas, which can be administered earlier.

Kampo formulas that are used for the treatment of influenza have been reported to inhibit the growth of influenza viruses in cultured cells (Mantani et al., 1999; Nomura et al., 2019; Hou et al., 2020). Although the relationship between inhibition of influenza virus replication in cultured cells and clinical efficacy is not entirely clear, the therapeutic effect of Kampo formulas may be elicited by suppression of the ability of the virus of replicate in the human respiratory tract. In addition, Lian hua qing wen, which contains Ephedrae herba and is one of the therapeutic agents used for influenza viruses in traditional Chinese medicine, has been shown i*n vitro* experiments to have an inhibitory effect on SARS-CoV-2 infection and has also been sown to be effective for COVID-19 in a clinical setting, (Su et al., 2020; Hu et al., 2021). Therefore, we investigated the effects of Kampo formulas on SARS-CoV-2 infection *in vitro* experiments.

Since commercial Kampo formulas contain additives and the types and amounts of additives are not uniform, we used the same concentrations of Kampo formulas without additives in this study. We found that virus infection was inhibited by the Kampo formulas Maoto, Saikokeishito, and Senkyuchachosan but not by the Kampo formulas Bakumondoto and Hochuekkito. We also examined the viral inhibitory effects of crude drugs as constituents of the Kampo formulas that had strong inhibitory effects on SARS-CoV-2 infection. Ephedrae herba had the strongest inhibitory effect on SARS-CoV-2 infection. Among the six crude drugs examined, Ephedrae herba, Paeoniae radix, Scutellariae radix, and Glycyrrhizae radix had the strongest inhibitory effects in that order.

Ephedrae herba showed direct inactivating effects on virus particles as well as on infected cells. Previous studies have shown that tannins, which are components of Ephedrae herba, inhibit influenza virus infection (Mantani et al., 1999) and that tannins extracted from plants such as persimmon have inactivating effects on various viruses (Ueda et al., 2013). In the present study, it was also thought that tannins in Ephedrae herba may have direct inhibitory effects on virus infection. However, in the infection experiments to investigate the suppressive effects of crude drugs, on virus infection, it was found that Ephedra herba had a suppressive effect even under the condition of an m.o.i. of 10, in which all cells were infected at once, and no virus particle formation process was involved. Therefore, Ephedrae herba may have both a direct inactivation effect on virus particles and a suppressive effect on infection of cells, suggesting a combined virus suppression mechanism.

However, it was found that Kampo formulas containing Ephedrae herba had little direct inactivation effect on virus particles. The contents of Ephedrae herba in the Kampo formulas used in the virus direct inactivation test (20 mg/ml) were 6.45 mg/ml in Maoto, 3.33 mg/ml in Kakkonto, and 2.22 mg/ml in Shoseiryuto. These concentrations were higher than the concentration of Ephedrae herba tested as a crude drug (1.25 mg/ml), which was found to be effective in the virus particle direct inactivation test. Furthermore, when the IC_50_ values of Kampo formulas containing Ephedrae herba and Ephedrae herba alone were compared on the basis of the content of Ephedrae herba, the effects of all Kampo formulas were weaker than the effect of Ephedrae herba alone. These results suggest that the combination of Ephedrae herba with other crude drugs in Kampo formulas has a weaker antiviral effect than that of Ephedrae herba. Ephedra herba has various side effects, and Kampo formulas are shown to contain crude drugs that alleviate the side effects of the strong Ephedra herba (Odaguchi et al., 2019). The reduced suppressive effect of Ephedra herba on virus infection may mean that its side effects on the human body are reduced.

Among the Kampo formulas that do not contain Ephedrae herba, Saikokeishito and Senkyuchachosan showed efficacy. These Kampo formulas have been reported to inhibit influenza virus *in vitro* (Shirayama et al., 2016; Nomura et al., 2019), and the Ministry of Health, Labour, and Welfare has approved Saikokeishito for treatment of influenza. Therefore, it would not be surprising if these Kampo formulas also have inhibitory effects on SARS-CoV-2 infection. Furthermore, viral inhibitory effects of Glycyrrhizae radix, Paeoniae radix, and Scutellariae radix as crude drugs contained in these Ephedrae herba-free Kampo formulas were confirmed. It was shown in an *in vitro* study that rhizoma had an inhibitory effect on influenza virus infecion (Nomura et al., 2019), but it had no inhibitory effect on SARS-CoV-2 infection.

Glycyrrhizae radix is a crude drug in many Kampo formulas. All of the Kampo formulas used in this study contained Glycyrrhizae radix (Maoto: 9.7%, Saikokeishito: 9.1%, Kakkonto: 11%, Shomakakkonto: 12.5%, Shoseiryuto: 11%, Senkyuchachosan: 7.5%, Bakumondoto: 7.4%, Hochuekkito: 6.3%; Table 1). Although a suppressive effect of Glycyrrhizae radix alone on SARS-CoV-2 infection was shown in a previous study (van de Sand et al., 2021), not all of the Kampo formulas containing Glycyrrhizae radix showed suppressive effects on virus infection, and some Kampo formulas showed no effect at all. Therefore, other crude drug components in the Kampo formulas may weaken the effect of Glycyrrhizae radix. Alternatively, the amount of Glycyrrhizae radix in the Kampo formulas may have been too small to reach the threshold for suppression of virus infection.

Paeoniae radix was suggested to be effective against SARS-CoV-2 infection by molecular modeling predictions (Ma et al., 2020). It was also shown to be effective against influenza virus infection in cell infection experiments (Ho et al., 2014). Scutellariae radix is one of the crude drugs contained in Saikokeishito, which was effective against SARS-CoV-2 infection. In addition, Baicalin, a component of Scutellariae radix, has been shown to have an inhibitory effect on SARS-CoV-2 infection in cell and animal experiments (Song et al., 2021). In the present study, Glycyrrhizae radix, Paeoniae radix, and Scutellariae radix showed inhibitory effects on SARS-CoV-2 infection, but their effects were inferior to the effect of Ephedrae herba.

## Conclusion

We performed *in vitro* experiments to determine the inhibitory effects on SARS-CoV-2 infection of Kampo formulas and their constituent crude drugs that are used to treat respiratory symptoms including influenza and common cold symptoms. We found that Maoto among the Kampo formulas and Ephedrae herba among the crude drugs had the strongest inhibitory effects on SARS-CoV-2 infection. Some other Kampo formulas and crude drugs also showed suppressive effects on virus infection. Although further analysis and evidence are needed, Kampo formulas might contribute to the treatment of COVID-19.

## Data Availability

The original contributions presented in the study are included in the article/Supplementary Material, further inquiries can be directed to the corresponding author.
